# Supplementary material for: Mechanical forces control the valency of the malaria adhesin VAR2CSA by exposing cryptic glycan binding sites
Source: PLoS Comput Biol. 2023 Dec 20;19(12):e1011726. doi: 10.1371/journal.pcbi.1011726 (PMC10786402; doi:10.1371/journal.pcbi.1011726)
Supplement: Attachment — Submitted filename: var2csa_response_letter.pdf [file pcbi.1011726.s005.pdf]

Dear editor,

We want to thank both reviewers for their valuable comments which have greatly improved our manuscript. We are very glad to learn that both reviewers find the manuscript very interesting and insightful for the biophysical community. Please find below our reply to their questions. Additional simulation analyses and changes we made to the manuscript are indicated here and also highlighted in the revised manuscript.

Reviewer #1: The authors analyze the molecular mechanism behind the shear enhanced activation of the binding between the VAR2CSA protein, which is produced by erythrocites infected with the malaria parasite Plasmodium falciparum, and the CSA receptor located on the placenta of pregnant women. The results suggest a catch-bond-like mechanism, but instead of increasing the lifetime of the bond between protein and ligand tensile force provides an additional two minor binding sites for the CSA receptor, which consists mainly of a dodecameric chain, hence increasing the valency of the protein-receptor interaction. This mechanism is supported by the observation in force probe simulations that the major binding site in VAR2CSA opens but the ligand remains bound. Such opening exposes two additional cryptic binding sites, which the authors show are large enough to accommodate each one additional CSA dodecameric glycan chain. The authors use a combination of equilibrium simulations (with no external tensile force) to test the stability of the VAR2CSA structure, which also contained segments that the authors had to model, force probe simulations with pulling at constant velocity, and computational docking where dodecameric glycan chains were docked to the cryptic sites that became exposed in the force probe simulations. The docking was then also followed by equilibrium simulations. The authors also performed various analyses, such as the number of contacts between protein and ligand along the simulation, and energetic calculations using the force field terms. Overall, the work is well done and will be of interest to the biophysical community.

I have a few minor comments, which are aimed at improving clarity and adding more quantification to the analysis:

1) Calculation of contacts: In the methods it says that the tool gmx mindist was used. I do not understand between what the minimum distance is calculated. Is it the minimum distance between two residues or the distance between two atoms? What is the distance cutoff used to define that two residues/atoms are forming a contact?

REPLY:

The minimum distance is between two atoms and the distance cutoff was 4.5 Å. This has been clarified in the revised manuscript (contact analysis methods section, lines 261–262).

2) In Figure 3D the claim is that the number of contacts decreases with opening distance and with simulation time. This is very difficult to tell by just looking at the plot. I think that the authors should calculate the Pearson's linear correlation coefficient, at least between number of contacts and opening distance. (Hint: in case the authors use xmgrace they may be aware that this can be done with: Data -> Transformations -> Regression)

REPLY:

We want to clarify that in Figure 3D the claim is that the number of contacts decreases –but by a little amount– with opening distance. This is a quantitative indication that opening

occurred earlier than sugar dissociation, i.e. the number of sugar-protein contacts never reached a zero value. We added this clarification to the revised manuscript. In addition we carried out the linear regression analysis suggested by the reviewer which further highlights this trend (result included in the caption of figure 3D). Changes made to the manuscript are highlighted in lines 394–400.

3) Bottom paragraph of page 11 discusses energetic calculations and the Coulombic attraction between the binding site and the glycan chain. Have the authors performed a hydrogen bond analysis between VAR2CSA and the glycan? For example, there are hydrogen bonds between the adhesin FimH (located at the tip of bacterial fimbria) and mannose, which are important for the stability of the bound conformation. I suspect that the hydrogen bonds between ligand and protein can provide a physical explanation for the larger Coulombic attraction between ligand and protein than between the two sub-regions. Can the authors identify examples of key persistent hydrogen bonds between VAR2CSA and CSA (major binding site) in the 200-ns long equilibrium simulations? Often, a contact is defined as persistent when it is formed in at least 66% of the simulation frames (excluding, e.g., the first 10 ns or so until the system has equilibrated), but it is up to the authors what criteria or cutoffs are best suited.

REPLY:
Following the reviewer's advice, we have carried out a hydrogen bond analysis between VAR2CSA and the glycan (see new figure 5D and text lines 289–297 and 455-458). In fact, multiple residues engage in strong and prevalent hydrogen bonds with the CSA glycan. However, unlike mannose, CSA contains negatively charged sulphate groups and such moieties establish very strong ionic pairs with the positively charged residues of VAR2CSA. For these reasons, in our analysis, we also took into account the ionic pairs. Accordingly, we made the distinction between the two types of interactions, i.e. hydrogen bonds in which uncharged residues participated and ionic pairs in which charged residues got involved. Both of them contribute to the stability of the bound conformation. In the discussion, we also comment on the difference between CSA and mannose.

Other minor comments:

4) Bottom of page 2 Introduction: "They are connected by four complex inter-domain regions (ID1 - ID3) ...". The list looks like it is only three. They should be all listed.

REPLY: They are all listed now (line 93).

5) Similarly, "... six Duffy-like binding domains (DBL1 - DBL6) ...", but then it talks about "DBL2X" and "DBL4 epsilon". I think they should be listed with their names when they are introduced.

REPLY: DBL domains are also listed with the complete names now (lines 90–91).

6) Page 6 sixth line: "domain portion of the major binding site that got exposed ..." This becomes clear after reading the Results, but it is a bit confusing. The authors may want to

write: "domain portion of the major binding site that is observed to become exposed in the force probe simulations".

REPLY: We rewrote this sentence following the suggestion of the referee.

7) At the end of the first paragraph at page 6, I had to read it many times to understand this part of the sentence: "..., which was not zero when their distance got below a distance of 13 nm". Maybe something like "..., which was non-zero when their distance was below 13 nm".

REPLY: We rewrote this sentence according to the reviewer's advice.

8) In section "Force opens the core region of tethered VAR2CSA", I think that it is not really necessary to repeat the methods such as the speed of 0.2 m/s and that "20 conformations were extracted from the equilibrium MD simulations". This information can probably be shortened while referring the reader to the Methods for details.

REPLY: We have shortened this section, leaving out the methodological details and referring to them in the methods section.

9) In "Conclusions", I got confused how many ligands in total were docked to the minor (cryptic) sites. It states: "Opening causes the exposure of two cryptic CSA binding sites. This enables VAR2CSA to stably bind a second CSA chain in addition to the one tethered to the major binding site". Is one additional CSA chain bound at the same time to both cryptic sites, or should it read: "This enables VAR2CSA to stably bind two additional CSA chains"?

REPLY: It is not. Each additional chain binds separately to each cryptic binding site and in addition to the chain that is bound to the main binding site. We reformulated this sentence to avoid the confusion (lines 596–598).

10) Last sentence of "Conclusions" should read (my additions or changes are in square brackets) "..., which can [be] directly tested by experiments and which may be relevant [to] the understanding of the malaria infection and [to] the development ...".

REPLY: we adopted the reviewer additions.

11) Second sentence of Author summary: "Malaria is caused [by] infection [with] the Plasmodium parasite, ..."

REPLY: Corrected.

12) Page 3 third paragraph, it should be "fimbrial adhesin" instead of "fimbrian adhesin".

REPLY: Corrected.

13) "helped VAR2CSA maintain" instead of "maintaining".

REPLY: Corrected.

14) The article needs to be proofread for other misspellings and typos.

REPLY: We have proofread the manuscript and corrected other misspellings and typos. We thank the reviewer for spotting them.

Reviewer #2: In the manuscript titled “Mechanical forces control the valency of the malaria adhesin VAR2CSA by exposing cryptic glycan binding sites”, Roessner et al. delve into the molecular mechanism governing the enhanced adhesion of Plasmodium falciparum-infected erythrocytes to the placenta under shear flow conditions. The adhesion process is coordinated by the VAR2CSA protein, contributing significantly to pregnancy-associated malaria. The authors employ a set of simulation methodologies to reveal how mechanical forces can instigate significant conformational changes in VAR2CSA, unveiling additional binding sites and increasing its binding affinity from a monovalent to a multivalent state. The insights gathered from this study lay the foundation for the development of targeted vaccines against placental malaria.

Overall, the manuscript is well written, and the simulations employ cutting-edge methodologies. Constructing the structure of this complex was monumental and deserves acknowledgment as a significant achievement, meritorious of publication in its own right. The system, comprising approximately 700,000 atoms, underwent combined simulations of 2 microseconds, utilizing equilibrium molecular dynamics simulations. This facilitated a thorough examination of the structure's stability.

However, I have a few reservations regarding the stability of the structural model:

1. The increasing number of contacts over time in Figure 2D might indicate that the model structure hasn’t reached stability and might not be in its native conformation.
2. The high RMSD in Figure 2C could be masking large scale instability in some domains.

To address these concerns, I suggest performing an RMSD analysis for each protein domain independently, allowing for an in-depth assessment of the stability of individual domains. This approach could also pinpoint which sections of the protein complex are experiencing greater motion, thereby identifying potential areas of instability. Also, the authors could use the same approach to check two protein domains at the time, helping to identify motion between protein domain pairs.

REPLY:
To specifically address observation 1, we further inspected the number of contacts between the protein and the sugar . We computed the average ± standard deviation to check for possible drifts (see corrected figure 2D). In fact, there is an increase in the number of contacts. We attribute this slight increment to the larger flexibility of the modelled part of the chain (see Figure 2E). Nevertheless, we think the drift is rather small when we compare it with the variation in the number of contacts across different simulation replicas (standard deviation) and with the initial value.

In addition, we followed the reviewer's advice and performed an RMSD analysis for each domain separately (see figure S1 in the revised manuscript). As predicted by the reviewer, this approach helped us to pinpoint the domains that exhibit lower/larger deviations from the initial conformation. As expected, the DBL domains were the most stable ones, deviating less from the initial conformation (with RMSDs below 10 Angstrom). The inter-domains (ID)

and the NTS domain displayed larger deviations, although ID2a showed RMSDs comparable to those of the DBL domain. In any case, the RMSD converged and fluctuated around rather constant values in almost all cases, particularly for the domains engaging in interactions with the CSA sugar chains, i.e. NTS, DBL2X, DBL4e, and ID2a.

Finally, to check the motions between domain pairs, we computed the inter-domain number of contacts (see figure S2 in the revised manuscript). The figure highlights the overall preservation of the initial number of contacts, but giving clues on specific pairs which displayed a low variability at the interface (such as the DBL1X–DBL2X or the DBL3e–DBL4e pairs) or cases in which the variability was higher (e.g. NTS–DBL1X).

Overall, these extra analyses helped us to confirm further that the system reached stable equilibrium conformations and accordingly that they could be used as starting conformations for the force-probe MD simulations. We expand on these new analyses in the results of the revised manuscript (see lines 341–359).

Additionally, the manuscript includes a mechanical study utilizing steered molecular dynamics simulations. The size of the system and the number of replicas are admirable. However, I would like to propose an enhancement:

The discussion surrounding the analysis in Figure 3, especially 3D and 3E, could gain depth with the inclusion of a force propagation analysis. Given that one of the manuscript's authors has previously developed strategies for such analyses, incorporating this would provide a richer understanding of the geometric aspects of the mechanical bond, currently best represented by Figure 4A.

REPLY:
We thank the reviewer for the suggestion. In the revised manuscript, we carried out force distribution analysis. In fact, this analysis helped us to discern specific regions which were mechanically disrupted upon opening and to identify the way mechanical stress redistributes upon the force-induced opening of the protein. The analysis also appears useful to predict residue pairs as candidates for cysteine cross-linking experiments. This analysis is shown in new figures 4 of main text and S3 of supporting information. Inclusions related to this new analysis appear in the methods (lines 298–317), results (lines 406–425), and discussion (lines 588–590).

Furthermore, the discussion contrasting zipping versus shearing motion deserves more comprehensive coverage within the paper. This mechanism appears to be a pivotal finding and thus, a more elaborate discussion would greatly enrich the readers' understanding. This is especially pertinent as this mechanism has been identified in various other systems. The difference between both zippering and shearing has been also identified within a single protein complex by examination of how tethering geometry impacts unbinding mechanics.

REPLY:
In the revised manuscript we provide a more comprehensive coverage on the competition between zipping versus shearing motion. In addition to the two originally cited studies, we found five more works which examined how tethering geometry impacts both unfolding of

single protein domains and unbinding of protein complexes (see seven references 69–75 in revised manuscript). We apologise for overlooking these other studies earlier. We now contextualise our results in the light of the common theme that emerges from all these studies. In brief, we delve into the notion that the point of application of force and the subsequent orientation the molecule adopts with respect to the force vector dictate the mechanical resistance of biomolecules. This can result in pathways offering strong resistance to unfolding or unbinding, if multiple bonds have to simultaneously be broken, or pathways offering weak resistance, if bonds can be broken in sequence one by one. We also emphasise this is not a mere experimental construct but a key aspect of how mechanosensitive biomolecules regulate key biological processes. See the changes made to the manuscript in lines 535–545 of the discussion section.

In closing, I am a great fan of the work, and I hope that my suggestions help to improve it even further.

REPLY:
Thank you very much. Yes they have helped to improve the manuscript indeed.
